# Segmental arterial mediolysis of left gastric artery: a case report and review of pathology

**DOI:** 10.1186/1472-6890-13-26

**Published:** 2013-10-29

**Authors:** Azra Tabassum, Sanaz Sasani, Adeeb J Majid, Christopher Henderson, Neil D Merrett

**Affiliations:** 1 Department of Surgery, Liverpool Hospital, Liverpool, NSW 2170, Australia; 2 Department of Anatomical Pathology, South Western Area Pathology Service, Liverpool Hospital, Liverpool, NSW 2170, Australia; 3 School of Medicine, University of Western Sydney, Sydney, NSW, Australia

**Keywords:** Segmental arterial mediolysis, Haemorrhage, Pathology, Aneurysm, Vasculitis

## Abstract

**Background:**

Segmental arterial mediolysis (SAM) is a rare non inflammatory vascular disease that can present with massive haemorrhage, which may lead to death without prompt surgical intervention.

**Case presentation:**

A 60 years old Aboriginal female presented with life threatening, spontaneous intra-abdominal bleeding requiring an emergency laparotomy. The source of bleeding was found to be ruptured left gastric artery. A total gastrectomy was performed as a damage control procedure. A staged Roux-en-Y oesophago-jejunostomy with Hunt Lawrence pouch reconstruction was undertaken thirty six hours later. Histopathological findings revealed evidence of non-inflammatory segmental vascular damage with microaneurysm, consistent with segmental arterial mediolysis.

**Conclusion:**

Prompt resuscitation and surgical intervention can decrease the morbidity and mortality in this rare clinical entity.

## Background

Segmental Arterial Mediolysis (SAM) is an uncommon non-inflammatory and non-atherosclerotic vascular disease first described in adult autopsy specimens by Slavin and Gonzalez in 1976 [1,2]. Cases of similar morphology in epicardial coronary arteries of newborn infants had been reported before that in 1949 by Gruenwald [3].

Slavin initially used the term Segmental Mediolytic Arteritis to describe the entity, however a decade later suggested SAM as a more appropriate descriptive term for this process in view of the absence of any inflammatory aetiology, and a lack of clinical and laboratory evidence of vasculitis in affected patients [4].

SAM characteristically involves small to medium sized muscular abdominal and visceral arteries, but pulmonary [2,5] and cerebral [6] cases are also reported. Both genders are equally affected with predominance in the middle aged and the elderly [7-11].

A vasospastic aetiology has been suggested for the pathogenesis of SAM [7,11,12]. Pathologically SAM involves an injurious and reparative phase. The injurious phase commences with mediolysis and separation of media from adventitia [4]. Lysis occurs segmentally in the media, sparing the intima. This focal loss of muscular wall manifests as arterial dilatation with progression resulting in arterial gaps and /or arterial dissection.with the arterial gaps bridged by fibrin and granulation tissue. These can enlarge by detachment of residual connections to the wall and develop vascular aneurysms.

The injurious phase lesions may lead to dissection between the outer media and the adventitia. Two types of dissection are described; the initial dissecting hematoma/bleeding from the gap, and secondary dissection occurring in the reparative phase of SAM. The bleeding in the latter is derived from the fragile vessels in the granulation tissue which fills the defect between outer media and the adventitia disrupting over time, resulting in the haematoma and dissection. Stenosis and narrowing results from overgrowth reparative tissue with extension over the intima forming plaques. The lumen may then be further narrowed by thrombosis with occlusion [1,3,4,12].

There are several identifiable non-specific angiographic patterns which are suggestive of SAM, but pathology remains as the definite diagnostic gold standard. Patients can be treated by vascular embolization or surgical resection. The nature and course of the disease can be quite variable, with subclinical as well as acute presentations which may lead to delayed symptomatic dissections or arterial lesions similar to fibromuscular dysplasia [4]. Due to the relative scarcity of reports increased awareness is required to diagnose and treat this rare condition.

## Case presentation

A 60- year-old Aboriginal female, presented to our emergency department following collapse at home. She was on Warfarin for paroxysmal atrial fibrillation and mitral regurgitation and her past medical history included hypertension, hypercholesterolemia, depression and cerebro vascular accident without any residual effect.

On presentation, she was agitated; her heart rate was 96/minute and systolic blood pressure was 70 mm of mercury. Clinical examination showed a distended abdomen with generalized tenderness. Laboratory investigation revealed Haemoglobin of 48 g/L, White blood count of 19.3 × 10/L and International Normalised Ratio (INR) of 1.7. Venous blood gases showed PH of 7.3, and a lactate of 6.03.

She was resuscitated with intravenous fluids, red blood cells and fresh frozen plasma. She had a cardiac arrest during resuscitation and Cardio Pulmonary Resuscitation was performed with good outcome. An urgent laparotomy was performed for apparent intra- abdominal bleeding.

Laparotomy showed 3 litres of free intraperitoneal blood and a large haematoma involving the lesser curve, upper body of stomach and the gastro oesophageal junction. The haematoma was expanding with active bleeding from the left gastric artery and its branches. Local measures to control the bleeding in this unstable patient failed and a total gastrectomy had to be performed.

Damage control principles were applied due to hemodynamic instability and reconstruction deferred with stapling of her oesophageal stump. An abdominal vacuum assisted closure (VAC™) dressing was applied and she was transferred to the Intensive care unit, where she was stabilised and her coagulopathy corrected.

A Roux-en-Y oesophago- jejunal anastomosis with Hunt - Lawrence pouch reconstruction and a feeding jejunostomy was undertaken thirty six hours later. Five days later, a gastrograffin swallow demonstrated anastomotic integrity; she was commenced on a diet and discharged 14 days after admission.

**Figure 1:**
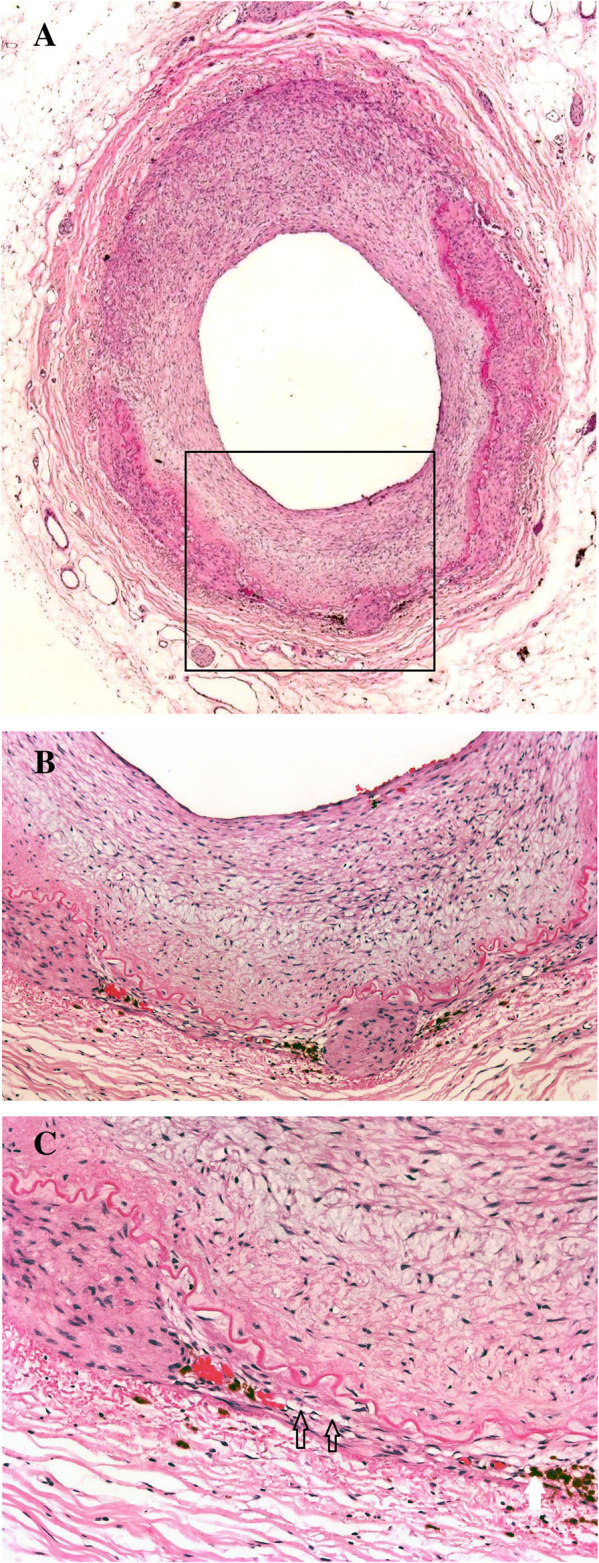
**Mediolysis of the media at low and high (×100) magnification (black arrows). A**. Note changes consistent with reparative phase with granulation tissue extending over the intima of arterial wall islands. **B** and **C** The black arrows demarcate mediolysis on higher magnification. The white arrow points to brown pigmentation of hemosiderin deposition. ×40 - ×100. Note the focus of intact muscle cells in area of total medial muscle loss.

**Figure 2:**
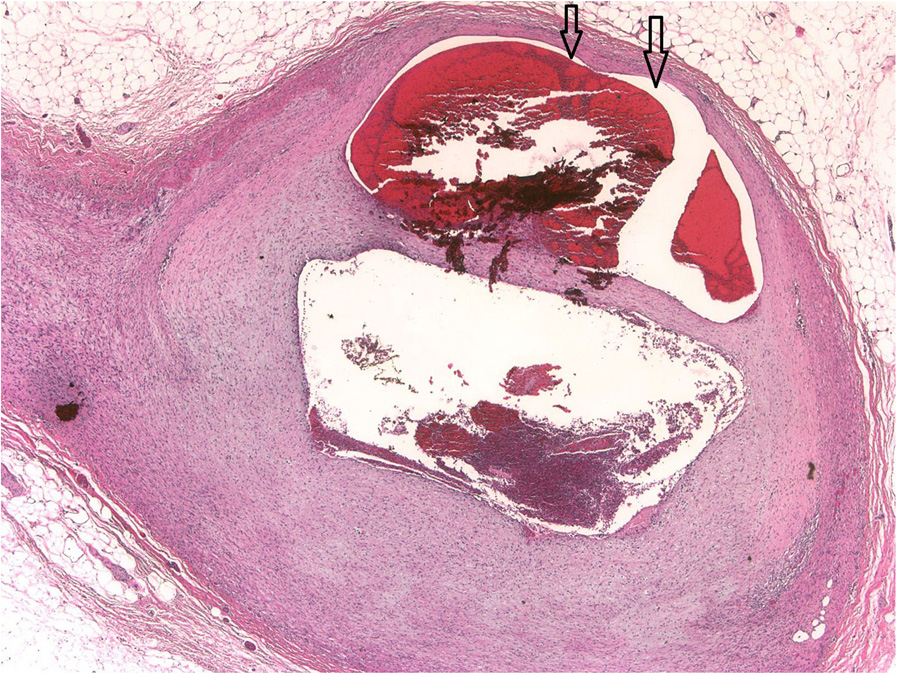
The cross section of a dissecting hematoma and the development of microaneurysm in keeping with late stage lesion ×20.

Histological evaluation of the gastrectomy specimen and extensive sampling of the vessels revealed the diagnostic clues. Figures 1A, B and 2 show SAM in the reparative phase with significant segmental disruption of the media by pronounced fibromyxoid proliferation and marked expansion of the intima in the moderate-sized arteries, with granulation tissue filling gaps and extending over the intima of arterial wall islands. In other areas of the arteries, there is often marked attenuation of media with gap formation only prevented by retained internal elastic and intima (Figures 1A&B). The lesions showed only scanty inflammation characterized by some occasional sparse lymphocytes and collection of haemosiderin containing histiocytes (Figure 1C). Focally, there was abrupt disruption of media with recoil of the internal elastic lamina, fibromyxoid tissue herniation into the adventitia and microaneurysmal appearances (Figure 2). Occasional small arteries showed intimal thickening, internal elastic lamina fragmentation, and attenuation of media. There was no evident feature of fibrosis and scarring.

Follow-up Computerised Tomography (CT) Mesenteric Angiogram scan 3 months later showed no significant vascular structure abnormality. Twelve months later, the patient remains asymptomatic.

## Discussion

SAM is rare vascular condition which is an unusual cause of unexplained intra-abdominal bleeding. This was first reported in 2004 where an emergency hemicolectomy was performed for an acutely bleeding inferior mesenteric artery [13]. The acute presentation is that of a significant intra-abdominal bleed leading to hypovolemic shock. But SAM may also manifest as acute intestinal ischemia consequent to thrombosis or dissection of the affected artery [14-20]. Mortality rate of about 50% has been reported in this acute phase [13].

This report highlights the presentation of SAM with acute bleeding requiring hemodynamic stabilisation and haemostasis. Radiological investigations such as Computerised Tomography (CT) and angiography may reveal patterns consistent with SAM including single or multiple aneurysms, dissection, stenosis and occlusion, but these may not be feasible in the unstable patients and definitive diagnosis is usually only arrived at histology [20].

SAM mainly involves the splanchnic and cerebral territories [6]. The most common sites are celiac axis, splenic, superior mesenteric, renal, and inferior mesenteric arteries. The involvement of cerebral vasculature is less frequent, affecting a younger population [9]. Usually, the immediate small branches of the affected arteries also show features of SAM [4].

Vasospasm is thought to be the underlying aetiological agent based on morphologic and ultra-structural features [9,10,12]. Factors including hypoxia, exogenous vasopressors, pulmonary hypertension, central nervous system lesions can initiate the dysfunction of endothelial paracrine system causing intense vasoconstriction.

Iatrogenic aetiology has been proposed with evidence implicating alpha-1 adrenergic receptors and Beta-2 agonists after similar lesions were induced in dogs after administration of ractopamine [12]. In rural industries, ractopamine is used for weight gain and as a partitioning agent. Australian regulatory authorities ban the use of ractopamine in beef and sheep farming but it is used in pork farming. Our patient was not taking any adrenergic agonists to treat her co morbidities, and she denies any known pork meal in the weeks leading up to her presentation.

A regional selectivity of endothelial responses to the stimuli may explain the fact that SAM is restricted to certain arterial beds [10]. Endothelin-1 (ET-1) has been implicated in the pathogenesis as it is overexpressed in SAM Endothelial cells can synthesize and rapidly release this pressor agent which can cause smooth muscle constriction and promote endothelial and fibroblastic proliferation [10]. ET-1 also potentiates the activity of norepinephrine and other pressor agents [7].

Pathologically SAM involves two phases, injurious and reparative. The injurious phase commences with mediolysis and separation of media from adventitia [4]. The lytic process involves two types of degenerative changes of the smooth muscle: vacuolization of cells presenting as clear cellular contents and contraction of the smooth muscle cells with nuclear pyknosis and cytoplasmic granular eosinophilia [10]. These changes typically occur in the outer media with characteristic segmental distribution. The intima and internal elastic lamina are spared [4]. The lytic areas contain cellular remnants within oedema-like fluid that may cause bowing of the wall [6]. This focal loss of muscular wall manifests as arterial dilatation on angiography. Progression results in transmedial mediolysis with loss of intima creating arterial gaps bridged by fibrin. These can enlarge by detachment of residual connections to the wall and develop vascular aneurysms which is the most frequent angiographic feature of SAM [4,9,10]. The smaller saccular aneurysms might decrease in size or resolve, while the larger ones may thrombose or bleed requiring radiologic or surgical intervention. Multiple aneurysms with segmental distribution lead to a string of beads appearance on angiography [4].

The injurious phase lesions may lead to dissection between the outer media and the adventitia. Two types of dissection are described; the initial dissecting hematoma/bleeding from the gap, and secondary dissection occurring in the reparative phase of SAM. The bleeding in the latter is derived from the fragile vessels in the granulation tissue which fills the defect between outer media and the adventitia disrupting over time, resulting in the haematoma and dissection.

Stenosis and narrowing results from overgrowth reparative tissue with extension over the intima forming plaques. The lumen may then be further narrowed by thrombosis with occlusion. These features might be noted at the onset of SAM or weeks-months later leading to collateral circulation and recanalization [4,9]. Older healed lesions have been described as intimal thickening, distortion and reduplication of elastic lamina and neovascularization of media as was also noted in our case [6].

Venous angiopathy has been reported in the medium to large size veins adjacent to the arteries affected by SAM. Described features include haphazard patchy loss of media (moth eaten appearance), plication of overlying intima and prominent endothelial cells predisposing to thrombus formation. In venopathy the endothelial and smooth muscles cells are stained with ET-1 whilst arterial pathology demonstrates ET-1 in the adventitia and in the granulation tissue of the reparative phase. Further studies are required to fully elucidate ET-1 potential role in pathogenesis [7].

Thus angiographic findings may be correlated to the pathophysiology of SAM with arterial dilatation, single/multiple aneurysm, dissecting hematoma, arterial stenosis, and finally arterial occlusion [4,7,9].

As there are clinical and radiological similarities between SAM and Fibromuscular dysplasia (FMD) [1,4,6,7,9,10,20,21] a relationship between the conditions has been proposed. Lei suggested that SAM is a variant of FMD [22] while Slavin believed that SAM could be a precursor of FMD with the reparative fibrosis transforming to arterial FMD [1,4,6]. However, there are differences between SAM and FMD in age and gender distribution, sites, and presentation [9] with FMD affecting primarily young to middle aged women with a predisposition for the renal arteries [9,20,21]. Potentially, asymptomatic cases of SAM without dissections and minor or no gaps could evolve to medial fibroplasias or perimedial dysplasias, the two most common types of FMD. SAM however usually affects older age groups where muscular-stromal connections are poor resulting in larger gaps and bleeding. FMD however, occurs in younger individuals with intact connections explaining the different presentation with ischemia and hypertension due to reparative stenosis [4].

Autoimmunity has also been proposed as an aetiological factor in the pathogenesis of SAM. Immunoglobulin G, immunoglobulin M, and C3a deposits have been demonstrated in both intact and involved vascular components in SAM. Moreover, an association of autoimmune conditions such as Crohn’s, systemic lupus erythematous, and Graves’s disease has been detected [9] and cystic medial degeneration has also been described coexisting with SAM [23].

A combination of pathologic features and clinical findings can assist to differentiate SAM from other conditions including various vasculitides, connective tissue disorders, infections (mycotic aneurysms and endocarditis), atherosclerosis, and inherited vascular defects [20]. The discrimination from systemic vasculitis is particularly critical since the therapeutic corticosteroids and immunosuppressive agents are contraindicated in SAM as they can induce iatrogenic sepsis [1,2,20]. Immune mediated vasculitis is usually diagnosed based on trans-arterial inflammation and fibrinoid necrosis. Finally, dissecting aneurysms [1,4] of other types are recognized by intimal tear and rupture of vasovasorum which is a different pathogenesis from SAM [20]. There were no histological findings supporting a diagnosis of vasculitis in our case.

## Conclusion

In summary, SAM can present with sudden onset of intra-abdominal bleeding, retroperitoneal bleeding, or brain haemorrhage and the diagnosis should be considered in cases of unexplained intra-abdominal haemorrhage. Should the patient’s condition permit investigation angiography can reveal several non-specific patterns which can be suggestive of SAM. A definitive diagnosis is based on pathologic evaluation of the involved vessels which requires extensive sampling and thorough assessment. After acute treatment, investigations should be performed to exclude other vascular abnormalities. Effective resuscitation and surgical intervention using damage control principles can achieve good survival outcomes. An increased awareness is required to diagnose and treat this rare clinical entity.

## Consent

Written informed consent was obtained from the patient for publication of this case report and accompanying images. A copy of the written consent is available for review by the Editor-in-Chief of this journal.

## Abbreviations

SAM: Segmental arterial mediolysis; CT: Computerised tomography; ET-1: Endothelin 1; FMD: Fibromuscular dysplasia.

## Competing interests

The authors declare that they have no competing interests.

## Authors’ contributions

AT, SS, AJM were involved with patient management and preparation of the manuscript. CH was involved in the preparation of the histological information and images, NDM conceived the report and participated in coordinating and drafting of the report giving final of the version to be published. All authors have reviewed and approved the final version.

## Pre-publication history

The pre-publication history for this paper can be accessed here:

http://www.biomedcentral.com/1472-6890/13/26/prepub
